# Efficacy of acupuncture for sciatica: study protocol for a randomized controlled pilot trial

**DOI:** 10.1186/s13063-020-04961-4

**Published:** 2021-01-07

**Authors:** Fang-Ting Yu, Guang-Xia Ni, Guo-Wei Cai, Wen-Jun Wan, Xiao-Qing Zhou, Xiu-Li Meng, Jin-Ling Li, Jian-Feng Tu, Li-Qiong Wang, Jing-Wen Yang, Hai-Yang Fu, Xin-Chang Zhang, Jing Li, Yan-Fu Wang, Beng Zhang, Xiao-Hui Zhang, Hao-Lin Zhang, Guang-Xia Shi, Cun-Zhi Liu

**Affiliations:** 1 International Acupuncture and Moxibustion Innovation Institute, Beijing University of Chinese Medicine, Beijing, 100029 China; 2 Acupuncture Research Center, School of Acupuncture-Moxibustion and Tuina, Beijing University of Chinese Medicine, Beijing, 100029 China; 3 Nanjing University of Chinese Medicine, Nanjing, 210023 China; 4 Department of Acupuncture, Union Hospital, Tongji Medical College, Huazhong University of Science and Technology, Wuhan, 430022 China; 5 Department of Rehabilitation, The Central Hospital of Wuhan, Tongji Medical College, Huazhong University of Science and Technology, Wuhan, 430014 China; 6 Shenzhen Hospital, Beijing University of Chinese Medicine, Shenzhen, 518100 China; 7 Pain Medicine Center, Peking University Third Hospital, Beijing, 100191 China; 8 Affiliated Hospital of Nanjing University of Chinese Medicine, Nanjing, 210029 China; 9 Department of Traditional Chinese Medicine, Peking University of Third Hospital, Beijing, 100191 China; 10 Department of acupuncture, Dongzhimen Hospital Affiliated to Beijing University of Chinese Medicine, Beijing, 100700 China

## Abstract

**Background:**

Acupuncture is widely used for pain diseases while evidence of its efficacy for sciatica is insufficient. We aim to explore the feasibility and efficacy of acupuncture with different acupoint selecting strategies for sciatica induced by lumbar disc herniation.

**Methods:**

This is a multicenter, three-arm, patient-assessor-blinded randomized controlled pilot trial. Ninety patients will be assigned randomly into 3 groups including disease-affected meridians (DAM) group, non-affected meridians (NAM) group, and sham acupuncture (SA) group in a 1:1:1 ratio. The trial involves a 4-week treatment along with follow-up for 22 weeks. The primary outcome is the change of leg pain intensity measured by the visual analogue scale (VAS) from baseline to week 4 after randomization. Secondary outcomes include functional status, back pain intensity, and quality of life. Adverse events will also be recorded.

**Discussion:**

The results will inspire the optimal acupuncture strategy for sciatica and help establish a better design as well as power calculation for a full-scale study.

**Trial registration:**

ChiCTR2000030680 (Chinese Clinical Trial Registry, http://www.chictr.org.cn, registered on 9 March 2020).

**Supplementary Information:**

The online version contains supplementary material available at 10.1186/s13063-020-04961-4.

## Background

Sciatica is characterized by radiating leg pain along the course of the sciatic nerve sometimes accompanied by back pain and neurological deficits [1]. The prevalence ranges from 1.2 to 43% globally based on controversial definitions [2]. Lumbar disc herniation is the leading cause of 85% of patients with sciatica [3]. Sciatica affects daily life and productivity and consumes more health resources when compared to low back pain [4].

Conservative treatments are the first-line options for sciatica [5, 6]. Medicine and epidural steroid injection are commonly used although long-term benefits are uncertain and side effects (e.g., headache and dizziness) or complications (e.g., epidural hematoma) occur sometimes [7–9]. Most pain and related disabilities could resolve in weeks [5], but up to 30% of patients were reported with pain lasting for 1 year or longer [10]. Therefore, long-term effective and safe conservative treatments might be potential solutions.

Acupuncture has been widely used for pain diseases. Meta-analysis indicated that acupuncture had a persistent effect on 4 chronic pains which decreased only 15% after 1 year [11]. However, high-quality evidence remains scarce for acupuncture treating sciatica [12, 13]. Acupoints play a key role in treatment that different acupoint selecting strategies could generate discrepant effects [14, 15]. To optimize acupuncture strategy for sciatica, this pilot study will evaluate feasibility and efficacy of acupuncture with different acupoint selecting methods. The hypothesis is that acupuncture on disease-affected meridians might have better effects. Reliable data will be used to develop a better design including sample size calculation for a further randomized controlled trial (RCT).

## Methods/design

### Study design

**Fig. 1:**
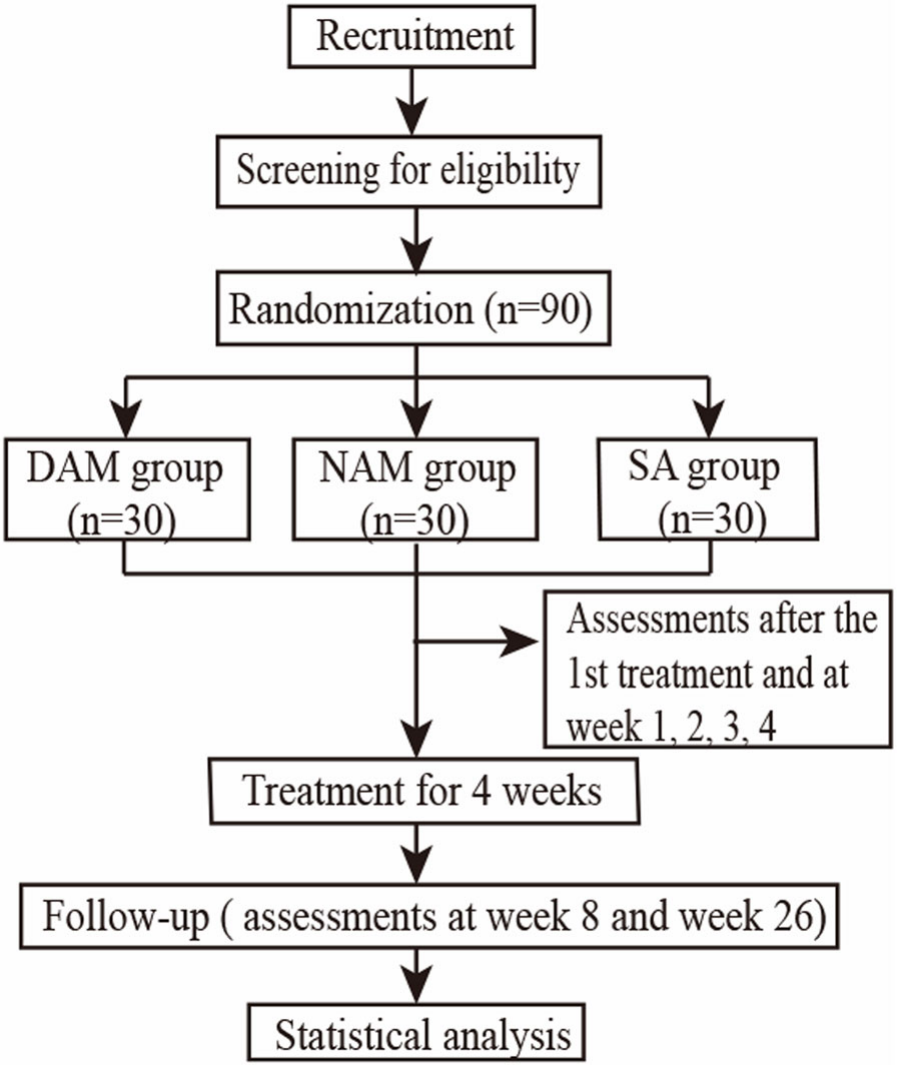
Flowchart of the trial procedure. DAM, disease-affected meridians; NAM, non-affected meridians; SA, sham acupuncture

The detailed study process is illustrated in Fig. 1. We design this multicenter, parallel-group, patient-assessor blinded RCT following the Consolidated Standards of Reporting Trials (CONSORT) and the Standards for Reporting Interventions in Clinical Trials of Acupuncture (STRICTA) guidelines [16, 17]. The trial will be conducted in outpatient departments of 6 hospitals in 4 cities in China, including the Department of acupuncture, Union Hospital, Union Hospital, Tongji Medical College, Huazhong University of Science and Technology; the Department of acupuncture, Affiliated Hospital of Nanjing University of Chinese Medicine; the Department of Rehabilitation, the Central Hospital of Wuhan, Tongji Medical College, Huazhong University of Science and Technology; the Department of acupuncture, Shenzhen Hospital, Beijing University of Chinese Medicine; the Department of pain medicine center and traditional Chinese medicine, Peking University Third Hospital; and the Department of acupuncture, Dongzhimen Hospital Affiliated to Beijing University of Chinese Medicine. The Research Ethics Committee of Beijing University of Chinese Medicine has approved the study protocol (version 2.0, 13 January 2020) with an approval number of 2020BZHYLL0105. It was registered at the Chinese Clinical Trial Registry (http://www.chictr.org.cn) on 9th March 2020 with the registration number of ChiCTR2000030680. The protocol is reported in accordance with the Standard Protocol Items (SPIRIT) (Additional file 1).

### Patients

There will be 6 centers conducting this study to ensure recruitments where many patients visit doctors for sciatica every day. Eligible patients will be recruited from outpatient clinics through advertisements on posters, newspapers, and WeChat. To promote recruitment as well as compliance, all treatment during the study will be offered freely.

#### Inclusion criteria

The study will include both male and female patients (1) aged between 18 and 70 years; (2) having symptoms of sciatica for 1 month or longer from the onset, diagnosed as unilateral radiating leg pain below the knee with positive straight-leg raise test or at least one neurological deficit symptoms as paraesthesia, muscle weakness, or reflex abnormalities. The imaging evidence of lumbar disc herniation is requested on either magnetic resonance imaging (MRI) or computed tomography (CT); (3) leg pain intensity for 40 mm or more measured by the visual analogue scale (VAS) [18].

#### Exclusion criteria

Patients will be excluded if they meet any of the following criteria: (1) patients who have or are suspected to have severe spinal diseases (e.g., cauda equina syndrome) or progressive neurological symptoms (e.g., progressive muscle weakness); (2) patients with sciatica caused by other conditions than lumbar disc herniation; (3) patients who have undergone surgery for lumbar disc herniation within the past 6 months; (4) patients who plan to have spinal surgery or other interventional therapies during the first 4 weeks of the trial; (5) patients continually taking antiepileptic medication, antidepressant medication, opioids or corticosteroids; (6) patients who have cardiovascular, liver, kidney, or hematopoietic system diseases, mental health disorders, or other severe coexisting diseases (e.g., cancer); (7) patients who are pregnant, breastfeeding, or planning to conceive during the trial; (8) patients who received acupuncture therapy within the past 6 months.

#### Dropout criteria

Patients will be removed if they are unwilling to continue participation. The reasons for dropouts will be recorded.

### Randomization

An independent statistician will generate the random sequence by the central block randomization method using IBM SPSS Statistics, version 21.0 (International Business Machines Corporation, China). Eligible patients will be assigned randomly into the disease-affected meridians (DAM) group, non-affected meridians (NAM) group, and sham acupuncture (SA) in a 1:1:1 ratio. An independent researcher in charge of informing assignment will implement the allocation schedule through a centralized telephone randomization procedure. To ensure the allocation concealment, acupuncturists will make a call to ask for the information of assignment before the first treatment. The independent researcher will send messages containing allocation details immediately. Before randomization, trial information will be provided to each eligible patient. Researchers (doctors) will get written informed consent from eligible patients who are willing to participate.

### Blinding

**Fig. 2:**
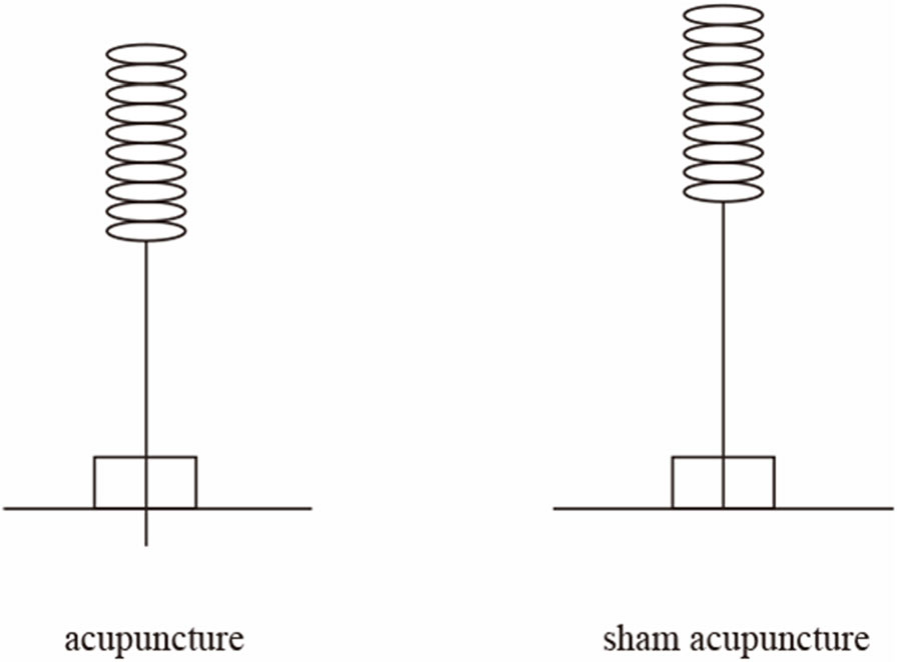
Schematic diagram of acupuncture and sham acupuncture

Patients, outcome assessors, and statistician will be blinded to the assignment. Differences between groups present mainly at point selections and acupuncture performance. Blunt-tipped needles for blinding will be similar to conventional needles. Adhesive foam pads will be put on treating points to cover the difference in acupuncture performance (Fig. 2).

### Interventions

**Fig. 3:**
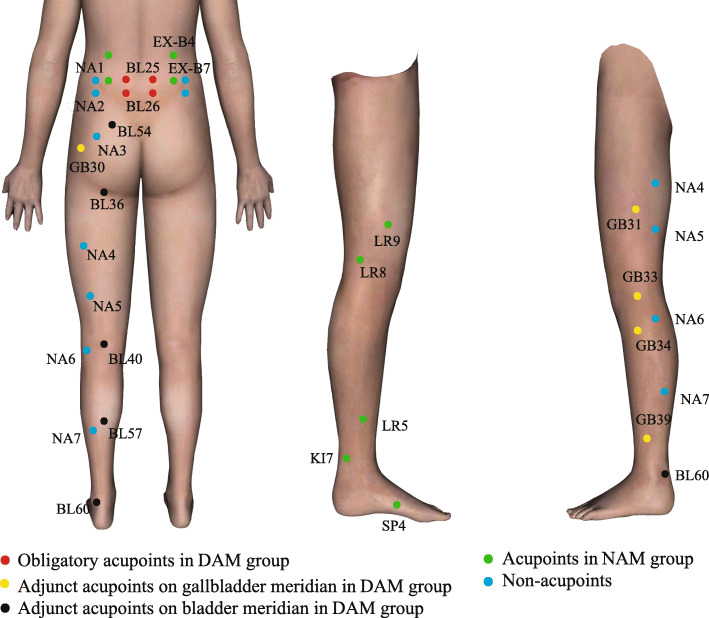
Locations of acupoints and non-acupoints. DAM, disease-affected meridians; NAM, non-affected meridians; SA, sham acupuncture

All patients will receive advice for managing sciatica in daily life such as remaining active, using a hard bed, or losing weight. Based on the assignment, they will receive 12 sessions of 30-min treatments over 4 weeks (ideally 3 times a week with an interval of 1 or 2 days). Acupoints are predefined based on experienced acupuncturists’ consensus and literature review [19]. Locations of acupoints are all according to the WHO Standard Acupuncture Locations shown in Tables 1 and 2 and Fig. 3 [20]. Patients could ask for Celebrex (Pfizer Pharmaceutical Co., Ltd) from researchers with guidance, and the use will be recorded in detail.

**Table 1:** Location of acupoints in DAM group

Acupoints	Location
Dachangshu (BL25)	In the lumbar region, at the same level as the inferior border of the spinous process of the fourth lumbar vertebra (L4), 1.5 cun lateral to the posterior median line.
Guanyuanshu (BL26)	In the lumbar region, at the same level as the inferior border of the spinous process of the fifth lumbar vertebra (L5), 1.5 cun lateral to the posterior median line
Huantiao (GB30)	In the buttock region, at the junction of the lateral one third and medial two thirds of the line connecting the prominence of the greater trochanter with the sacral hiatus.
Fengshi (GB31)	On the lateral aspect of the thigh, in the depression posterior to the iliotibial band where the tip of the middle finger rests, when standing up with the arms hanging alongside the thigh.
Xiyangguan (GB33)	On the lateral aspect of the knee, in the depression between the biceps femoris tendon and the iliotibial band, posterior and proximal to the lateral epicondyle of the femur.
Yanglingquan (GB34)	On the fibular aspect of the leg, in the depression anterior and distal to the head of the fibula.
Xuanzhong (GB39)	On the fibular aspect of the leg, anterior to the fibula, 3 cun proximal to the prominence of the lateral malleolus.
Zhibian (BL54)	In the buttock region, at the same level as the fourth posterior sacral foramen, 3 cun lateral to the median sacral crest.
Chengfu (BL36)	In the buttock region, at the midpoint of the gluteal fold.
Weizhong (BL40)	On the posterior aspect of the knee, at the midpoint of the popliteal crease.
Chengshan (BL57)	On the posterior aspect of the leg, at the connecting point of the calcaneal tendon with the two muscle bellies of the gastrocnemius muscle.
Kunlun (BL60)	On the posterolateral aspect of the ankle, in the depression between the prominence of the lateral malleolus and the calcaneal tendon.

One “cun” is defined as the width of the interphalangeal joint of patient’s thumb

**Table 2:** Location of acupoints in NAM group

Acupoints	Location
Yaoyan (EX-B7)	In the lumbar region, at the same level as the inferior border of the spinous process of the fourth lumbar vertebra (L4), 3.5 cun lateral to the posterior median line.
Pigen (EX-B4)	In the lumbar region, at the same level as the inferior border of the spinous process of the first lumbar vertebra (L1), 3.5 cun lateral to the posterior median line.
Yinbao (LR9)	On the medial aspect of the thigh, between the gracilis and the sartorius muscles, 4 cun proximal to the base of the patella.
Ququan (LR8)	On the medial aspect of the knee, in the depression medial to the tendons of the semitendinosus and the semimembranosus muscles, at the medial end of the popliteal crease.
Ligou (LR5)	On the anteromedial aspect of the leg, at the center of the medial border (surface) of the tibia, 5 cun proximal to the prominence of the medial malleolus.
Fuliu (KI7)	On the posteromedial aspect of the leg, anterior to the calcaneal tendon, 2 cun superior to the prominence of the medial malleolus.
Gongsun (SP4)	On the medial aspect of the foot, anteroinferior to the base of the first metatarsal bone, at the border between the red and white flesh.

One “cun” is defined as the width of the interphalangeal joint of patient’s thumb

#### DAM group

Semi-standardized treatment will be provided with acupoints on affected meridians in this group. There will be four obligatory acupoints and five adjunct acupoints. Obligatory acupoints are bilateral Dachangshu (BL25) and Guanyuanshu (BL26). Depending on the distribution of the pain, sciatica localized at the side of the affected leg will be treated with adjunct acupoints on gallbladder meridian including Huangtiao (GB30), Fengshi (GB31), Xiyangguan (GB33), Yanglingquan (GB34), and Xuanzhong (GB39). Sciatica localized at the back of the affected leg will be treated with adjunct acupoints on bladder meridian including Zhibian (BL54), Chengfu (BL36), Weizhong (BL40), Chengshan (BL57), and Kunlun (BL60). For patients with pain in both side and back, adjunct acupoints will be selected from the ten adjunct acupoints above based on the personal experiences of acupuncturists.

After strict disinfection of the skin around acupoints and acupuncturists’ hands using 75% alcohol, adhesive foam pads (10-mm diameter and 5-mm height) will be placed on all acupoints with patients in a prone position. Disposable stainless steel acupuncture needles (0.30 × 75 mm, Suzhou Huatuo Medical Instrument Co, Ltd) will be inserted in BL25 and BL26 on the affected side deeply to 40–70 mm. After performing twirling, lifting, and thrusting manipulations for about 10 s, patients are expected to have “de qi” sensation (a complex feeling including soreness, numbness, heaviness, distention and dull pain, etc.) radiating down to the affected leg. Other acupoints will be treated with needles (0.30 mm × 40/50/75 mm) inserted to normal depths to reach “de qi” sensation locally. All needles will be retained for 30 min.

#### NAM group

Nine acupoints on non-affected meridians will be used as follows: bilateral Yaoyan (EX-B7) and Pigen (EX-B4) and unilateral Yinbao (LR9), Ququan (LR8), Ligou (LR5), Fuliu (KI7), and Gongsun (SP4) on the affected leg. All acupoints will be treated by needles (0.30 × 40/50 mm) inserted into the skin with manipulations later to reach the “de qi” sensation. Other treatment settings are the same as those for the DAM group.

#### SA group

We select nine non-acupoints in this group with locations shown in Table 3. They are localized in the middle of the gallbladder meridian and bladder meridian as one selecting method for non-acupoints [21]. Blunt-tipped needles (0.30 × 25 mm) will be inserted only into the pad with no penetration into the skin. No “de qi” sensation is required in this group and other settings will be the same.

**Table 3:** Location of non-acupoints in SA group

Non-acupoints	Location
NA 1 (bilateral)	In the lumbar region, 2.5 cun beside Dachangshu (BL25), in the middle of gallbladder meridian and bladder meridian.
NA 2 (bilateral)	In the lumbar region, 2.5 cun beside Guanyuanshu (BL26), in the middle of gallbladder meridian and bladder meridian.
NA 3	In the middle of Zhibian (BL54) and Huantiao (GB30) acupoints on the affected leg.
NA 4	10 cun above the popliteal crease, in the middle of gallbladder meridian and bladder meridian on the affected leg.
NA 5	5 cun above the popliteal crease, in the middle of gallbladder meridian and bladder meridian on the affected leg.
NA 6	In the middle of Weizhong (BL40) and Yanglingquan (GB34) acupoints on the affected leg.
NA 7	In the middle of Chengshan (BL57) and Waiqiu (GB36) acupoints on the affected leg.

One “cun” is defined as the width of the interphalangeal joint of patient’s thumb

### Outcomes

The primary outcome is defined as the change of leg pain intensity on VAS from baseline to week 4 measuring pain over the prior 24 h. The VAS presents as a 0–100 mm ruler with 0 representing no pain and 100 representing unbearable pain. The specific score will be determined by the distance from 0 to the patient’s mark.

Secondary outcomes include VAS for leg pain and back pain at other time points, Oswestry Disability Index (ODI) [22], the Sciatica Frequency and Bothersomeness Index (SFBI) [23], 36-item Short Form Health Survey (SF-36) [24], straight leg raise test [25], painDETECT questionnaire [26], global perceived recovery on 7-point Likert self-rating scale [27], the Credibility/Expectancy Questionnaire [28], blinding assessment and medicine use. The ODI plays a role in giving a subjective percentage score of function level for patients with low back pain through examining perceived disability in 10 activities of daily living. The SFBI measures the frequency and symptom severity with scores ranging from 0 to 24 respectively. SF-36 is used to assess the quality of life on eight aspects including physical functioning, bodily pain, role limitations due to physical health problems, personal or emotional problems, emotional well-being, social functioning, energy/fatigue, general health perceptions, and a single item that indicates perceived change in health. PainDETECT questionnaire is used to discern the variation of neuropathic pain as one common method. A 7-point Likert self-rating scale is used to assess the recovery compared to the onset with options from “completely recovered” to “worse than ever.” The Credibility/Expectancy Questionnaire is capable to assess the credibility and expectancy of patients. Maintenance of blinding will be determined by asking patients to report the group which they believe they had been assigned to. Categories and frequency of medicines use during the trial will be recorded in detail. Detailed arrangements of every outcome are shown in Table 4.

**Table 4:**
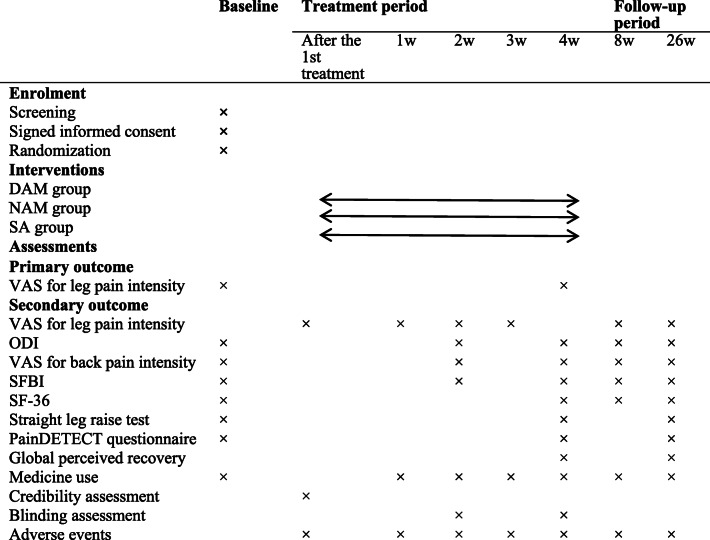
Schedule of recruitment, interventions, and assessments

*DAM* disease-affected meridians, *NAM* non-affected meridians, *SA* sham acupuncture, *w* week, *VAS* visual analogue scale, *ODI* Oswestry Disability Index, *SFBI* the Sciatica Frequency and Bothersomeness Index, *SF-36* 36-item Short Form Health Survey

Adverse events will be recorded, valued, and treated timely. Continuous investigation and reexaminations will be conducted until the end of the study. Independent clinical specialists who are blinded to the assignment will judge the severity and relationship between adverse events and acupuncture. Sever adverse events will be reported to the principal investigator and the data and safety monitor board (DSMB) within 24 h after occurrence to determine subsequent participation. The occurrence ratio of adverse events will be calculated at the end of the study.

### Data management

Data will be collected clearly and completely on case report forms (CRFs). All original data sources will be preserved including CRFs, informed consents, and inspection results. An independent data manager is in charge of the completed CRFs, he will be asked to input data doubly for proofreading. All data related to the trial will be saved for at least 5 years after publication. Readers will be permitted to access the original data by contacting the corresponding author. Information on patients will remain anonymous including name, age, and telephone number. An independent DSMB is established to ensure the integrity of the research data. The DSMB will review the progress and decide whether a premature closure is needed or not.

### Quality control

The study protocol has been reviewed and revised by experts of acupuncture, orthopedics, statistics, and methodology. All researchers who conduct the study will attend a series of training to promote the implementation. Data will be monitored on 3 levels by the head of sub-centers, researchers from the Beijing University of Chinese Medicine, and the DSMB pre-set for this study. The Research Ethics Committee of Beijing University of Chinese Medicine will audit the study on a regular basis independently.

### Sample size

As a pilot study, we determine to use a convenience sample instead of a sample size calculation. Ninety patients will be enrolled in this trial, 30 in each group. The data of this pilot trial will be applied to facilitate the calculation of the sample size for a further RCT.

### Statistical analysis

Continuous variables will be described by means and standard deviations or 95% confidence intervals when following a normal distribution. If not, the data will be shown by medians and interquartile ranges. Categorical variables will be described by frequencies and percentages.

All data will be analyzed following the intention-to-treat principle primarily. A per-protocol analysis will be used for primary outcome as sensitivity analysis covering patients who complete at least 10 treatment sessions without obvious violation. Analysis of variance (ANOVA), Kruskal-Wallis H test, or chi-square test will be used to compare the equilibrium between three groups at baseline. As for outcomes, one-way repeated measures ANOVA or Kruskal-Wallis H test will be applied for continuous variables and chi-square tests or Fisher’s exact test will be used for categorical variables as appropriate. Data analyses will be performed using IBM SPSS21.0 with the statistical significance of two-tailed *P* < 0.05 as exploratory.

## Discussion

Acupuncture might be a potential therapy for sciatica while methodological issues (e.g., lack of random sequence generation, allocation concealment, and blinding) appear frequently in previous studies. Two recent trials provided restricted evidence in favor of acupuncture but both were conducted in a single center with small sample sizes [29, 30]. Generalization of these study results might be limited. This pilot study will be conducted in 6 centers and will develop a full-scale study in order to address the issues.

The mechanism of sciatica may relate to the distortion of the nerve roots and effect of local inflammatory cytokines [1]. Acupuncture is known to exert an analgesic effect through inhibiting cytokine production and activate sympathetic nerve fibers to increase endogenous opioids [31, 32]. It may normalize default mode network activity and modulation of descending pain processing that help treat sciatica [33].

According to the Traditional Chinese Medicine theory, sciatica belongs to disorders of gallbladder meridian and bladder meridian. Acupuncturists tend to choose acupoints on disease-affected meridians in clinical practice which is expected to produce better effect. In our study, acupoints in the DAM group are all attached to the two affected meridians while those in the NAM group are on other meridians less related to sciatica. The comparative results will inspire the optimal acupuncture strategy for sciatica.

The feasibility and efficacy of acupuncture for sciatica will be evaluated in this study. Other strengths involve that it is a multicenter study designed following the Good Clinical Practice guideline [34]. Acupoints are selected based on literature and practical experiences from experts. Our limitations include that we use a subjective primary outcome, while VAS is the commonest method for measuring pain. Based on practical conditions, acupuncturists could not be blinded in this trial but communications will be limited to minimize the impact. Outcomes of this pilot study will be used as evidence for a further RCT subsequently.

## Trial status

Recruitment began on June 9, 2020, and was completed on September 27, 2020. This pilot study (protocol version 2.0, 13 January 2020) is expected to be finished by the end of March 2021.

## Amendments

### Change in study centers

Affected by the COVID-19, the Guang’anmen Hospital failed to get administrative support from government and was not permitted to recruit patients. To ensure recruitment and reach the target sample size, we had to add 3 other centers: the Union Hospital, Tongji Medical College, Huazhong University of Science and Technology; the Central Hospital of Wuhan, Tongji Medical College, Huazhong University of Science and Technology; and the Shenzhen Hospital, Beijing University of Chinese Medicine. Finally, 6 hospitals are conducting this pilot study. The final center was confirmed on 26 June, 2020.

### Change in inclusion criteria

We planned to enroll patients with acute, subcute, and chronic sciatica initially. However, as many patients chose to stay at home rather than visit doctors under the impact of the COVID-19 pandemic, we realized it could be very hard to enroll patients with acute sciatica. After discussions with experts, we finally decided to enroll patients with sciatica for 1 month or longer. This change was made on May 28, 2020.

### Change in exclusion criteria

The length of time for patients receiving acupuncture treatment previously was adjusted from 1 year to 6 months in exclusion criteria. This change was made based on advice from methodological experts who suggested that it would be enough to exclude the lasting effects of acupuncture in 6 months. This change was made on May 28, 2020.

### Change in secondary outcome

Based on suggestions from experts, we have added “the Sciatica Frequency and Bothersomeness Index” as an additional outcome to measure the frequency and bothersomeness for sciatica. It is commonly used in previous studies with good reliability [24]. This change was made on May 28, 2020.

## Supplementary Information

**Additional file 1.** SPIRIT 2013 Checklist: Recommended items to address in a clinical trial protocol and related documents.

## Data Availability

Data sharing is not applicable to this article as no datasets were generated or analyzed during the current study.

## Abbreviations

DAM: Disease-affected meridians
NAM: Non-affected meridians
SA: Sham acupuncture
RCT: Randomized controlled trial
CONSORT: Consolidated Standards of Reporting Trials
STRICTA: The Standards for Reporting Interventions in Clinical Trials of Acupuncture guidelines
SPIRIT: The Standard Protocol Items
DSMB: Data and safety monitor board
CRF: Case report form
MRI: Magnetic resonance imaging
CT: Computed tomography
VAS: Visual analogue scale
ODI: Oswestry Disability Index
SFBI: The Sciatica Frequency and Bothersomeness Index
SF-36: 36-item Short Form Health Survey
ANOVA: Analysis of variance
